# Common variation in *EMSY *and risk of breast and ovarian cancer: a case-control study using HapMap tagging SNPs

**DOI:** 10.1186/1471-2407-5-81

**Published:** 2005-07-19

**Authors:** Patrick R Benusiglio, Fabienne Lesueur, Craig Luccarini, Joan McIntosh, Robert N Luben, Paula Smith, Alison Dunning, Douglas F Easton, Bruce AJ Ponder, Paul D Pharoah

**Affiliations:** 1 Department of Oncology, University of Cambridge, Strangeways Research Laboratory, Worts Causeway, Cambridge CB1 8RN, UK; 2 Centre National de Genotypage, 2 rue Gaston Cremieux, CP 5721, 91057 Evry Cedex, France; 3 EPIC, University of Cambridge, Strangeways Research Laboratory, Worts Causeway, Cambridge CB1 8RN, UK; 4 Department of Genetic Epidemiology, University of Cambridge, Strangeways Research Laboratory, Worts Causeway, Cambridge CB1 8RN, UK

## Abstract

**Background:**

*EMSY *could be involved in low-level susceptibility to breast and ovarian cancer. Gene amplification is seen in a proportion of breast and ovarian tumours and correlates with poor prognosis in breast cancer patients. Furthermore, the EMSY protein silences a transcription activation domain in *BRCA2 *exon 3.

**Methods:**

We used a genetic association study design to determine if common genetic variation (frequency ≥ 5%) in *EMSY *was associated with breast or ovarian cancer risk in the British population. Haplotype tagging single-nucleotide polymorphisms (htSNPs) were selected from the HapMap database and genotyped using Taqman^® ^in two large study sets of white British women (n [breast set] = 2343 cases and 2284 controls, n [ovarian set] = 864 cases and 864 controls). HapMap data might be insufficient to tag genetic variation in *EMSY *comprehensively. We therefore screened the gene promoter and coding sequences with denaturing high performance liquid chromatography in order to identify additional SNPs that are most likely to be functional.

**Results:**

HapMap data on 22 SNPs show that 4 htSNPs tag 4 common haplotypes: rs2282611 (5'up t>g), rs4245443 (IVS7 g>a), rs2513511 (IVS16 a>g), rs2155220 (3'down c>t). We observed no association between any of the genotypes or associated haplotypes and breast or ovarian cancer risk. Seventeen out of the 18 remaining HapMap polymorphisms (94%) were well tagged by the 4 selected htSNPs (r^2^_s _> 0.8). Genotype frequencies for two further SNPs identified by screening and located near exon-intron boundaries, rs2508740 (IVS9 a>g) and rs11600501 (IVS10 c>t), were also similar in cases and controls. In order to simulate unidentified SNPs, we performed the leave-one-out cross-validation procedure on the HapMap data; over 95% of the common genetic variation was well represented by tagging polymorphisms. We are therefore likely to have tagged any common, functional variants present in our population.

**Conclusion:**

We found no association between common genetic variation in *EMSY *and risk of breast or ovarian cancer in two large study sets of white British women.

## Background

Breast and ovarian cancer are two of the most common causes of cancer in women in the United Kingdom (Office for National Statistics). Together, they account for about a third of all new cancer cases and a quarter of cancer deaths. Positive family history is a well-established risk factor for both diseases: the risk to first-degree relatives of a case is about 2 times the population risk [1-3]. Most of the excess familial risk associated with breast and ovarian cancer is likely to be genetic in origin [3,4]. However, only a small proportion of this risk is accounted for by known highly predisposing genes, *BRCA1 *and *BRCA2*, while the remainder might be explained by a combination of weakly predisposing alleles [5-10]. *EMSY *(*C11orf30*) is a novel gene that could be involved in low-level predisposition to breast and ovarian cancer [11]. Its recent discovery generated widespread interest [12,13]. The gene maps to chromosome 11q13, spans 103.3 kilobases and comprises 20 coding exons. *EMSY *is amplified in 12% of breast cancers and 17% of high-grade ovarian cancers and its amplification has been associated with an increased risk of relapse as well as decreased survival in breast cancer patients [11,14]. Furthermore, the EMSY protein silences the transcriptional activation potential of *BRCA2 *exon 3, a region deleted in a Swedish breast and ovarian cancer family [11,15].

The case-control study design is well suited to the identification of small-effect genes that are likely to underlie common, complex diseases such as breast or ovarian cancer: a difference in allele frequency is sought between affected individuals and unrelated controls [16]. Two approaches have been proposed. The traditional, hypothesis-driven approach is to investigate single-nucleotide polymorphisms (SNPs) in coding regions, since they are more likely to have a functional role and to influence directly the traits under study [17]. The alternative, indirect approach is to select a set of haplotype-tagging SNPs (htSNPs); htSNPs are informative polymorphisms that best characterize haplotype diversity and therefore genetic variation within the gene [18,19]. They serve as markers to detect associations between a particular region and diseases, whether or not the SNPs themselves have a functional effect [20,21]. It is not necessary to genotype all possible polymorphisms because the alleles of SNPs that are physically close to each other tend to be correlated with each other: they are in linkage disequilibrium (LD) [22]. The HapMap online database allows the indirect approach to be applied readily to many genes or regions [23]. By December 2004, the database held the genotypes of 90 individuals with northern and western European ancestry for over 850'000 SNPs.

We used a genetic association study design to determine if variation in *EMSY *was associated with breast or ovarian cancer risk. In order to have good power to detect small relative risks, we restricted our attention to common SNPs and haplotypes (frequency ≥ 5%). We first selected htSNPs using HapMap data. We also screened the gene promoter and coding regions in order to identify additional polymorphisms that are likely to be functional, as HapMap might be insufficient to tag genetic variation comprehensively. The selected SNPs were then genotyped in two large case-control sets (one breast cancer set and one ovarian cancer set) of white British women.

## Methods

### Patients and controls

Cases were drawn from the breast and ovarian arms of the SEARCH Cancer Study, an ongoing population-based study with cases ascertained through the East Anglia and West Midlands cancer registries in the United Kingdom [5,24]. All women diagnosed after 1990 with invasive breast cancer under the age of 70 years, or epithelial ovarian cancer under the age of 75 years, were eligible for inclusion. Approximately 65% of eligible breast cancer patients and 60% of ovarian cancer patients have enrolled in the study. Women taking part were asked to provide a 20-ml blood sample for DNA analysis and to complete a comprehensive epidemiological questionnaire. We carried out genotyping on sub-sets consisting of the first 2343 (breast cancer) and 864 (ovarian cancer) cases. Median age at diagnosis was 51 years for breast cancer cases (age range 25 to 69) and 55 years for ovarian cancer cases (age range 16 to 74). Two thousand two hundred and eighty-four and 864 controls were randomly drawn from the Norfolk component of the European Prospective Investigation of Cancer (EPIC), for the breast and the ovarian studies, respectively [25]. The EPIC-Norfolk cohort comprises 25,000 individuals resident in Norfolk (East Anglia), ages 45–74 years. The ethnic background of both cases and controls is similar, with over 98% being white Europeans. Ethical approval was obtained from the Anglia and Oxford Multicentre Research Committee and the Norwich Local Research Ethics Committee and informed consent was obtained from each patient.

### SNP identification and selection

We selected htSNPs from the HapMap database (, public releases up to September 2004) with the TagSNPs program [26], including 5 kilobases upstream and downstream the gene and aiming for a minimum r^2^_h _of 0.8. r^2^_h _is a measure of correlation between haplotypes defined by all SNPs and haplotypes defined by the selected htSNPs. At the time of selection, genotypes were only available for the Centre d'Etude du Polymorphisme Humain (CEPH) samples: these were collected in 1980 from people living in Utah with ancestry from northern and western Europe.

In order to screen the gene promoter and coding regions for polymorphisms, we performed denaturing high performance liquid chromatography (DHPLC) using the Wavemaker detection system (version 4.1, Transgenomics, Crewe, United Kingdom), followed by sequencing (3100 Genetic Analyser, Applied Biosystems, Warrington, United Kingdom) on genomic DNA from 48 random breast cancer cases. A 600-base pair putative promoter starting 500 base pairs upstream the gene was identified with gene2promoter , a program that allows for automated extraction of groups of promoters from a list of accession numbers or gene IDs.

### Genotyping

Genotyping was carried out using Taqman^® ^(Applied Biosystems) according to manufacturer's instructions. Primers and probes were supplied directly by Applied Biosystems except those for IVS9 a>g that were designed using Primer Express Oligo Design Software v2.0 (Applied Biosystems). Sequences are available on request. Reactions were carried out at 60°C in 384-well plates with cases and controls plated together. Each plate included 2 negative controls with no DNA and 12 samples duplicated on a separate quality control plate. Plates were read on the ABI Prism 7900 using the Sequence Detection Software (Applied Biosystems). Failed genotypes were not repeated.

### Statistical methods

For each SNP, deviation of genotype frequencies in controls from the Hardy-Weinberg equilibrium was assessed by a χ^2 ^test with one degree of freedom (df). Genotype frequencies in cases and controls were compared by a χ^2 ^test for heterogeneity (2df). Genotype-specific risks were estimated as odds ratios (OR) using standard cross-product ratio and confidence intervals were calculated using the variance of the log (OR), which was estimated by the standard Taylor expansion. A comparison of haplotype frequencies between cases and controls was carried out using the haplo.score routine implemented in S-plus [27]. Haplotypes with an estimated frequency of less than 5% were pooled. Haplo.score uses a likelihood that depends on estimated haplotype frequencies to test the statistical association between haplotypes and phenotype. It is based on score statistics, which provide both global tests and haplotype-specific tests [27]. The LDA program [28] was used to calculate pairwise LD for SNP pairs in the breast cancer study set. LDA is a Java-based program implementing the EM algorithm for pairwise LD analysis [28].

Power was determined using standard statistical methods [29]. We have over 90% power at the 1% significance level to detect a dominant allele with a frequency of 0.2 that confers a relative risk of breast cancer of 1.3 or a relative risk of ovarian cancer of 1.6. Power to detect recessive alleles at the 1% significance level is more limited: 59% for an allele with a frequency of 0.2 that confers a relative risk of breast cancer of 1.5 or 51% for an allele with a frequency of 0.3 that confers a relative risk of ovarian cancer of 1.5.

## Results

Genotypes for 22 common *EMSY *SNPs were available in HapMap, none of the SNPs were in coding regions. The working density was therefore of one SNP per 5 kilobases. The gene consisted of only one LD block [21]. There were 5 common haplotypes which constituted 92% of all the observed haplotypes and were tagged by 5 htSNPs: rs2282611 (5'up t>g), rs4245443 (IVS7 g>a), rs2513511 (IVS16 a>g), rs2155220 (3'down c>t) and rs7106446 (table 1). Taqman^® ^assays were successfully designed for the first four, but an assay could not be designed for rs7106446. There were no alternative SNPs with similar tagging properties. We were thus left with 4 htSNPs tagging 4 common haplotypes.

Genotyping success rate was over 92%. None of the genotype distributions in the controls differed significantly from those expected under Hardy-Weinberg equilibrium. There was no evidence that any of the SNPs is associated with breast (table 2) or ovarian cancer (table 3); genotype-specific OR were all close to unity with narrow confidence intervals. There was no association of genotype with age in controls and, as expected, age adjusted risks were close to the unadjusted risks (data not shown). The 4 htSNPs generated 5 common haplotypes in our population; the global test of association was not significant for breast cancer (P = 0.27) or for ovarian cancer (P = 0.93), nor were there any differences between cases and controls for the individual haplotype frequencies (Additional file: 1). The number of common haplotypes tagged by the 4 selected htSNPs differed between HapMap (n = 4) and our study (n = 5) because two rare HapMap haplotypes tagged by SNP rs1939468 were grouped into our fifth common haplotype (Additional file: 1).

**Figure 1:**
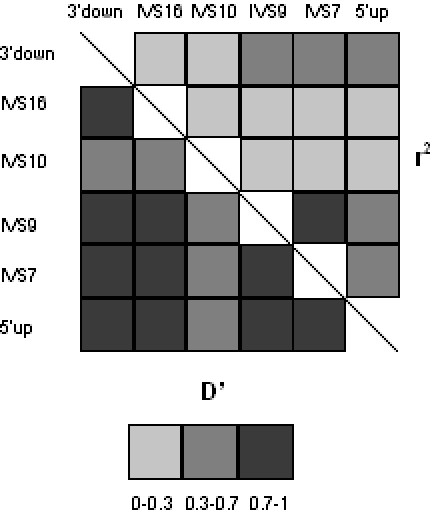
Linkage disequilibrium (LD). Pairwise (LD) measures of D' (left bottom half) and r^2 ^(right top half) for the six single-nucleotide polymorphisms (SNPs) genotyped in the breast and ovarian cancer study sets.

Screening of the promoter and coding regions revealed two further SNPs located near exon-intron boundaries, rs2508740 (IVS9 a>g) located 4 base pairs upstream exon 10 and rs11600501 (IVS10 c>t) located 14 base pairs upstream exon 11; neither of these were associated with breast or ovarian cancer risk (tables 2 and 3). At the time of study, there were four putative, non-validated coding SNPs mentioned in the dbSNP database : rs1954782, rs11822571, rs3753051 and rs1047196. We did not detect any of them. LD was strong (D' > 0.7) across pairs involving all SNPs except IVS10 c>t while IVS7 g>a and IVS9 a>g were in nearly perfect LD (r^2 ^= 0.94) (figure 1).

## Discussion

This is the first association study reporting results on *EMSY*, a gene of importance through its interaction with *BRCA2 *and its amplification status in tumours. We found no association between any of the *EMSY *genotypes or associated haplotypes and risk of breast or ovarian cancer in a white British population. We could have failed to observe a true association because of a Type II statistical error, but the large size of our study gives us high statistical power and strongly reduces the likelihood that our results are influenced by chance fluctuations in the case or control genotype frequency [30].

An alternative reason for failure to observe a true association could be that our set of tagging SNPs are poor markers of a true causal variant, which would either be one of the known SNPs in the gene or an as yet unidentified variant. In HapMap, common *EMSY *haplotypes were tagged by 5 SNPs. However, an assay for rs7106446 could not be designed and thus our htSNP set was suboptimal. Where a tagging SNP is used as a marker for a true disease-predisposing SNP the effective sample size is proportional to the bivariate correlation coefficient (r^2^) between the marker and causal SNPs [31]. r^2^_s _is the squared correlation coefficient between multi-locus haplotypes and individuals SNPs and is analogous to r^2^. In order to establish how well we had tagged the known SNPs with our set of tagging SNPs, we calculated r^2^_s _[26] between the 4 selected htSNPs and the 18 remaining HapMap polymorphisms. Seventeen (94%) SNPs were tagged with r^2^_s _> 0.8 but 1 SNP, rs7106446, was tagged with r^2^_s _= 0.46. Loss of power was therefore marginal for all HapMap SNPs except rs7601446; for a SNP tagged with r^2^_s _= 0.85, we had 89% power at the 1% significance level to detect a dominant allele with a frequency of 0.3 that confers a relative risk of ovarian cancer of 1.5.

It is also possible that we have not adequately tagged an unidentified, disease-predisposing SNP. Whole-gene resequencing across a sample population would be required to identify all existing polymorphisms and allow investigators to select htSNPs that tag all genetic variants. The HapMap project does not re-sequence the genome; it validates SNPs from the dbSNP public database, aiming for a density of polymorphisms that cover the whole of genetic variation across the human genome. Comprehensive tagging requires a genotyping density of around 1 SNP per 2.5 kb [32]. The 1 SNP per 5 kb density available for *EMSY *in HapMap might therefore be insufficient. In order to identify additional SNPs that are most likely to be functional [17], we screened the gene promoter and coding sequence with DHPLC, a technique with an estimated sensitivity of 94% [33]. Two SNPs near exon-intron boundaries were identified but neither was associated with breast or ovarian cancer.

We also assessed how well a set of htSNPs would have tagged any unidentified SNPs using a leave-one-out cross validation procedure on the HapMap data: each of the 22 known SNPs were dropped in turn and htSNPs selected from the remaining 21, thus simulating unidentified polymorphisms [32]. The ability of htSNPs to tag the dropped SNP was then evaluated by calculating r^2^_s _[26]. Mean r^2^_s _was 0.94. Twenty-one (95.4%) out of 22 dropped SNPs were tagged with r^2^_s _> 0.8, and only 1 (4.6%) was tagged with 0.4 < r^2^_s _< 0.8. Over 95% of the common genetic variation in *EMSY *should therefore be well represented by tagging polymorphisms. We are therefore likely to have tagged any common, functional variants present in our population. After this study was completed and the first version of the manuscript submitted, genotyping data in a white American population for rs3753051, a synonymous coding SNP in exon 19, were released in dbSNP. We were able to assess how this polymorphism was tagged by our set of SNPs as genotypes from the same individuals were also available for 5'up t>g, IVS9 a>g, IVS16 a>g and 3'down c>t; SNP rs3753051 was perfectly tagged (r^2 ^= 1) by 5'up t>g.

This study design can not exclude the involvement of a rare allele in predisposition to breast or ovarian cancer; for example, CHEK2*1100delC, has a frequency of around 1% and was recently shown to confer a two-fold increased risk of breast cancer [34]. Our study set would be too small to detect the effect of such an allele if it doubled the risk of ovarian cancer. Some authors have advocated the use of histopathologic or demographic data that subclassify individuals in order to identify homogeneous subsets for analysis [35]. In the absence of any main effect or strong biological rationale, we have not carried out subgroup analyses as much larger sample sizes would be required to obtain reliable results. The number of possible post-hoc, subgroup analyses is large and there is a strong possibility that one or more tests will be statistically significant simply by chance [36].

We are reporting results for a set of htSNPs selected from HapMap. We used genotypes for the CEPH samples to choose htSNPs. According to the HapMap website, it is unclear how accurately the CEPH samples reflect the patterns of genetic variation in people with northern and western European ancestry. Our results suggest that they correctly predict genetic variation in our white British population: allele frequencies in the breast study controls were similar to those obtained from HapMap (P = 0.57, 0.99, 0.88 and 0.85 for 5'up t>g, IVS7 g>a, IVS16 a>g and 3'down c>t, respectively), thus strengthening the argument for a widespread use of the database for htSNPs selection. A predisposing SNP might have a differential effect in another ethnic group via gene-gene or gene-environment interactions, although in a recent study of the genetic effects for 43 validated gene-disease associations across 697 study populations of various descents, Ioannidis *et al*. concluded that, even if frequencies of polymorphisms varied among populations, their biological impact on the risk for common diseases should be consistent across traditional ethnic boundaries [37,38].

## Conclusion

We saw no association between common SNPs in *EMSY *or their associated haplotypes with risk of breast or ovarian cancer in two large study sets of white British women.

## Competing interests

The author(s) declare that they have no competing interests.

## Authors' contributions

PRB carried out the experiments, performed the analyses and wrote the manuscript under the supervision of FL, BAJP and PDP. CL managed the genotyping process. JM and RL collected DNA from cancer cases and EPIC controls, respectively. PS contributed to the haplotype analyses. AD was the laboratory manager. DFE was the statistical advisor. All authors read and approved the final manuscript.

## Pre-publication history

The pre-publication history for this paper can be accessed here:



## Supplementary Material

Additional file 1 Click here for file

## Figures and Tables

**Table 1:** Haplotype-tagging single-nucleotide polymorphisms (htSNPs) selected from the HapMap database

dbSNP id	SNP name	Frequency	Location
rs2282611	5'up t>g	0.35	START-3136
rs4245443	IVS7 g>a	0.40	Intron 7 +40
rs2513511	IVS16 a>g	0.14	Intron 16 +1947
rs2155220	3'down c>t	0.45	END +3584

**Table 2:** Single-nucleotide polymorphisms (SNPs) in the breast cancer study set. Allele frequencies, genotype frequencies and genotype-specific risks in 2343 women with breast cancer and 2284 controls. OR, odds ratio; CI, confidence intervals; RAF, rare allele frequency; M/M, common homozygotes; M/m, heterozygotes; m/m, rare homozygotes; df, degrees of freedom.

SNP	Series	RAF	M/M n (%)	M/m n (%)	m/m n (%)	Number genotyped	P 2 df
5'up t>g	Cases	0.33	989 (45)	961 (44)	231 (11)	2181	
	Controls	0.32	1069 (47)	959 (42)	249 (11)	2277	0.42
	OR (95% CI)		1 (ref)	1.08 (0.96–1.23)	1.00 (0.82–1.22)		
IVS7 g>a	Cases	0.40	727 (36)	980 (48)	317 (16)	2024	
	Controls	0.40	803 (37)	1025 (47)	364 (16)	2192	0.51
	OR (95% CI)		1 (ref)	1.06 (0.92–1.21)	0.96 (0.80–1.15)		
IVS16 a>g	Cases	0.13	1629 (75)	502 (23)	36 (2)	2167	
	Controls	0.14	1690 (75)	526 (23)	42 (2)	2258	0.87
	OR (95% CI)		1 (ref)	0.99 (0.86–1.14)	0.89 (0.57–1.40)		
3'down c>t	Cases	0.44	638 (32)	986 (49)	399 (20)	2023	
	Controls	0.44	691 (31)	1073 (49)	440 (20)	2204	0.98
	OR (95% CI)		1 (ref)	1.00 (0.87–1.14)	0.98 (0.83–1.17)		
IVS9 a>g	Cases	0.39	760 (37)	988 (48)	299 (15)	2047	
	Controls	0.39	847 (39)	1009 (46)	343 (16)	2199	0.29
	OR (95% CI)		1 (ref)	1.09 (0.96–1.24)	0.97 (0.81–1.17)		
IVS10 c>t	Cases	0.05	1824 (91)	177 (9)	3 (0)	2004	
	Controls	0.04	1956 (91)	187 (9)	3 (0)	2146	0.99
	OR (95% CI)		1 (ref)	1.02 (0.82–1.26)	1.07 (0.22–5.32)		

**Table 3:** Single-nucleotide polymorphisms (SNPs) in the ovarian cancer study set. Allele frequencies, genotype frequencies and genotype-specific risks in 864 women with ovarian cancer and 864 controls. OR, odds ratio; CI, confidence intervals; RAF, rare allele frequency; M/M, common homozygotes; M/m, heterozygotes; m/m, rare homozygotes; df, degrees of freedom.

SNP	Series	RAF	M/M n (%)	M/m n (%)	m/m n (%)	Number genotyped	P 2 df
5'up t>g	Cases	0.31	346 (47)	315 (43)	69 (9)	730	
	Controls	0.32	392 (46)	369 (43)	92 (11)	853	0.65
	OR (95% CI)		1 (ref)	0.97 (0.79–1.19)	0.85 (0.60–1.20)		
IVS7 g>a	Cases	0.40	222 (37)	289 (48)	95 (16)	606	
	Controls	0.40	304 (36)	405 (48)	140 (16)	849	0.90
	OR (95% CI)		1 (ref)	0.98 (0.78–1.23)	0.93 (0.68–1.27)		
IVS16 a>g	Cases	0.14	479 (74)	164 (25)	6 (1)	649	
	Controls	0.13	652 (76)	188 (22)	13 (2)	853	0.22
	OR (95% CI)		1 (ref)	1.19 (0.93–1.51)	0.63 (0.24–1.66)		
3'down c>t	Cases	0.44	219 (30)	370 (51)	141 (19)	730	
	Controls	0.43	283 (33)	412 (48)	159 (19)	854	0.41
	OR (95% CI)		1 (ref)	1.16 (0.93–1.45)	1.15 (0.86–1.53)		
IVS9 a>g	Cases	0.39	319 (37)	398 (47)	134 (16)	851	
	Controls	0.39	327 (38)	402 (47)	133 (15)	862	0.97
	OR (95% CI)		1 (ref)	1.01 (0.82–1.25)	1.03 (0.78–1.37)		
IVS10 c>t	Cases	0.04	778 (92)	68 (8)	0 (0)	846	
	Controls	0.05	776 (91)	77 (9)	2 (0)	855	0.29
	OR (95% CI)		1 (ref)	0.88 (0.63–1.24)	-		

## References

[B1] Edmondson RJ, Monaghan JM (2001). The epidemiology of ovarian cancer. Int J Gynecol Cancer.

[B2] (2001). Familial breast cancer: collaborative reanalysis of individual data from 52 epidemiological studies including 58,209 women with breast cancer and 101,986 women without the disease. Lancet.

[B3] Pharoah PD, Ponder BA (2002). The genetics of ovarian cancer. Best Pract Res Clin Obstet Gynaecol.

[B4] Ponder BA (2001). Cancer genetics. Nature.

[B5] (2000). Prevalence and penetrance of BRCA1 and BRCA2 mutations in a population-based series of breast cancer cases. Anglian Breast Cancer Study Group. Br J Cancer.

[B6] Dite GS, Jenkins MA, Southey MC, Hocking JS, Giles GG, McCredie MR, Venter DJ, Hopper JL (2003). Familial risks, early-onset breast cancer, and BRCA1 and BRCA2 germline mutations. J Natl Cancer Inst.

[B7] Peto J, Collins N, Barfoot R, Seal S, Warren W, Rahman N, Easton DF, Evans C, Deacon J, Stratton MR (1999). Prevalence of BRCA1 and BRCA2 gene mutations in patients with early-onset breast cancer. J Natl Cancer Inst.

[B8] Easton DF (1999). How many more breast cancer predisposition genes are there?. Breast Cancer Res.

[B9] Pharoah PD, Antoniou A, Bobrow M, Zimmern RL, Easton DF, Ponder BA (2002). Polygenic susceptibility to breast cancer and implications for prevention. Nat Genet.

[B10] Gayther SA, Russell P, Harrington P, Antoniou AC, Easton DF, Ponder BA (1999). The contribution of germline BRCA1 and BRCA2 mutations to familial ovarian cancer: no evidence for other ovarian cancer-susceptibility genes. Am J Hum Genet.

[B11] Hughes-Davies L, Huntsman D, Ruas M, Fuks F, Bye J, Chin SF, Milner J, Brown LA, Hsu F, Gilks B, Nielsen T, Schulzer M, Chia S, Ragaz J, Cahn A, Linger L, Ozdag H, Cattaneo E, Jordanova ES, Schuuring E, Yu DS, Venkitaraman A, Ponder B, Doherty A, Aparicio S, Bentley D, Theillet C, Ponting CP, Caldas C, Kouzarides T (2003). EMSY links the BRCA2 pathway to sporadic breast and ovarian cancer. Cell.

[B12] King MC (2004). A novel BRCA2-binding protein and breast and ovarian tumorigenesis. N Engl J Med.

[B13] Livingston DM (2004). EMSY, a BRCA-2 partner in crime. Nat Med.

[B14] Rodriguez C, Hughes-Davies L, Valles H, Orsetti B, Cuny M, Ursule L, Kouzarides T, Theillet C (2004). Amplification of the BRCA2 pathway gene EMSY in sporadic breast cancer is related to negative outcome. Clin Cancer Res.

[B15] Nordling M, Karlsson P, Wahlstrom J, Engwall Y, Wallgren A, Martinsson T (1998). A large deletion disrupts the exon 3 transcription activation domain of the BRCA2 gene in a breast/ovarian cancer family. Cancer Res.

[B16] Risch NJ (2000). Searching for genetic determinants in the new millennium. Nature.

[B17] Tabor HK, Risch NJ, Myers RM (2002). Opinion: Candidate-gene approaches for studying complex genetic traits: practical considerations. Nat Rev Genet.

[B18] Rebbeck TR, Ambrosone CB, Bell DA, Chanock SJ, Hayes RB, Kadlubar FF, Thomas DC (2004). SNPs, haplotypes, and cancer: applications in molecular epidemiology. Cancer Epidemiol Biomarkers Prev.

[B19] Freedman ML, Penney KL, Stram DO, Le Marchand L, Hirschhorn JN, Kolonel LN, Altshuler D, Henderson BE, Haiman CA (2004). Common variation in BRCA2 and breast cancer risk: a haplotype-based analysis in the Multiethnic Cohort. Hum Mol Genet.

[B20] Cardon LR, Abecasis GR (2003). Using haplotype blocks to map human complex trait loci. Trends Genet.

[B21] Gabriel SB, Schaffner SF, Nguyen H, Moore JM, Roy J, Blumenstiel B, Higgins J, DeFelice M, Lochner A, Faggart M, Liu-Cordero SN, Rotimi C, Adeyemo A, Cooper R, Ward R, Lander ES, Daly MJ, Altshuler D (2002). The structure of haplotype blocks in the human genome. Science.

[B22] Pharoah PD, Dunning AM, Ponder BA, Easton DF (2004). Association studies for finding cancer-susceptibility genetic variants. Nat Rev Cancer.

[B23] Gibbs RA, Belmont JW, Hardenbol P, Willis TD, Yu F, Yang H, Ch'ang LY, Huang W, Liu B, Shen Y, Tam PK, Tsui LC, Waye MM, Wong JT, Zeng C, Zhang Q, Chee MS, Galver LM, Kruglyak S, Murray SS, Oliphant AR, Montpetit A, Hudson TJ, Chagnon F, Ferretti V, Leboeuf M, Phillips MS, Verner A, Kwok PY, Duan S, Lind DL, Miller RD, Rice JP, Saccone NL, Taillon-Miller P, Xiao M, Nakamura Y, Sekine A, Sorimachi K, Tanaka T, Tanaka Y, Tsunoda T, Yoshino E, Bentley DR, Deloukas P, Hunt S, Powell D, Altshuler D, Gabriel SB, Zhang H, Matsuda I, Fukushima Y, Macer DR, Suda E, Rotimi CN, Adebamowo CA, Aniagwu T, Marshall PA, Matthew O, Nkwodimmah C, Royal CD, Leppert MF, Dixon M, Stein LD, Cunningham F, Kanani A, Thorisson GA, Chakravarti A, Chen PE, Cutler DJ, Kashuk CS, Donnelly P, Marchini J, McVean GA, Myers SR, Cardon LR, Abecasis GR, Morris A, Weir BS, Mullikin JC, Sherry ST, Feolo M, Altshuler D, Daly MJ, Schaffner SF, Qiu R, Kent A, Dunston GM, Kato K, Niikawa N, Knoppers BM, Foster MW, Clayton EW, Wang VO, Watkin J, Gibbs RA, Belmont JW, Sodergren E, Weinstock GM, Wilson RK, Fulton LL, Rogers J, Birren BW, Han H, Wang H, Godbout M, Wallenburg JC, L'Archeveque P, Bellemare G, Todani K, Fujita T, Tanaka S, Holden AL, Lai EH, Collins FS, Brooks LD, McEwen JE, Guyer MS, Jordan E, Peterson JL, Spiegel J, Sung LM, Zacharia LF, Kennedy K, Dunn MG, Seabrook R, Shillito M, Skene B, Stewart JG, Valle DL, Jorde LB, Belmont JW, Chakravarti A, Cho MK, Duster T, Foster MW, Jasperse M, Knoppers BM, Kwok PY, Licinio J, Long JC, Marshall PA, Ossorio PN, Wang VO, Rotimi CN, Royal CD, Spallone P, Terry SF, Lander ES, Lai EH, Nickerson DA, Altshuler D, Bentley DR, Boehnke M, Cardon LR, Daly MJ, Deloukas P, Douglas JA, Gabriel SB, Hudson RR, Hudson TJ, Kruglyak L, Kwok PY, Nakamura Y, Nussbaum RL, Royal CD, Schaffner SF, Sherry ST, Stein LD, Tanaka T (2003). The International HapMap Project. Nature.

[B24] Dicioccio RA, Song H, Waterfall C, Kimura MT, Nagase H, McGuire V, Hogdall E, Shah MN, Luben RN, Easton DF, Jacobs IJ, Ponder BA, Whittemore AS, Gayther SA, Pharoah PD, Kruger-Kjaer S (2004). STK15 polymorphisms and association with risk of invasive ovarian cancer. Cancer Epidemiol Biomarkers Prev.

[B25] Day N, Oakes S, Luben R, Khaw KT, Bingham S, Welch A, Wareham N (1999). EPIC-Norfolk: study design and characteristics of the cohort. European Prospective Investigation of Cancer. Br J Cancer.

[B26] Stram DO, Haiman CA, Hirschhorn JN, Altshuler D, Kolonel LN, Henderson BE, Pike MC (2003). Choosing haplotype-tagging SNPS based on unphased genotype data using a preliminary sample of unrelated subjects with an example from the Multiethnic Cohort Study. Hum Hered.

[B27] Schaid DJ, Rowland CM, Tines DE, Jacobson RM, Poland GA (2002). Score tests for association between traits and haplotypes when linkage phase is ambiguous. Am J Hum Genet.

[B28] Ding K, Zhou K, He F, Shen Y (2003). LDA--a java-based linkage disequilibrium analyzer. Bioinformatics.

[B29] Armitage P, Berry G (1994). The size of a statistical investigation. Statistical Methods in Medical Research.

[B30] Cox DG, Hankinson SE, Kraft P, Hunter DJ (2004). No association between GPX1 Pro198Leu and breast cancer risk. Cancer Epidemiol Biomarkers Prev.

[B31] Zondervan KT, Cardon LR (2004). The complex interplay among factors that influence allelic association. Nat Rev Genet.

[B32] Ahmadi KR, Weale ME, Xue ZY, Soranzo N, Yarnall DP, Briley JD, Maruyama Y, Kobayashi M, Wood NW, Spurr NK, Burns DK, Roses AD, Saunders AM, Goldstein DB (2005). A single-nucleotide polymorphism tagging set for human drug metabolism and transport. Nat Genet.

[B33] Klein B, Weirich G, Brauch H (2001). DHPLC-based germline mutation screening in the analysis of the VHL tumor suppressor gene: usefulness and limitations. Hum Genet.

[B34] (2004). CHEK2*1100delC and Susceptibility to Breast Cancer: A Collaborative Analysis Involving 10,860 Breast Cancer Cases and 9,065 Controls from 10 Studies. Am J Hum Genet.

[B35] Rebbeck TR, Martinez ME, Sellers TA, Shields PG, Wild CP, Potter JD (2004). Genetic variation and cancer: improving the environment for publication of association studies. Cancer Epidemiol Biomarkers Prev.

[B36] Colhoun HM, McKeigue PM, Davey SG (2003). Problems of reporting genetic associations with complex outcomes. Lancet.

[B37] Cui J, Zhou X, Chazaro I, DeStefano AL, Manolis AJ, Baldwin CT, Gavras H (2003). Association of polymorphisms in the promoter region of the PNMT gene with essential hypertension in African Americans but not in whites. Am J Hypertens.

[B38] Ioannidis JP, Ntzani EE, Trikalinos TA (2004). 'Racial' differences in genetic effects for complex diseases. Nat Genet.

