# Supplementary material for: Common variation in EMSY and risk of breast and ovarian cancer: a case-control study using HapMap tagging SNPs
Source: BMC Cancer. 2005 Jul 19;5:81. doi: 10.1186/1471-2407-5-81 (PMC1185523; doi:10.1186/1471-2407-5-81)
Supplement: Additional file 1 [file 1471-2407-5-81-S1.doc]

**Table 4**

**Haplotypes.**

Haplotype-tagging single-nucleotide polymorphisms (htSNPs) 5'up t>g, IVS7 g>a, IVS16 a>g and 3'down c>t were genotyped in the breast and ovarian cancer study sets. rs7106446 is a htSNP for which a genotyping assay could not be designed; in HapMap, it splits haplotype 4 in two haplotypes with similar frequencies. Two rare HapMap haplotypes tagged by SNP rs1939468 are grouped into haplotype 5. Estimated haplotype frequencies are given for the breast (BC) and ovarian cancer (OC) study sets and for the HapMap (HMP) database. Haplotype individual p-values are given for the two study sets. Haplotype global test p-value (BC) = 0.27, haplotype global test p-value (OC) = 0.93.

| Hap. | 5’up  t>g | IVS7  g>a | rs  7106446 | rs  1939468 | IVS16  a>g | 3’down  c>t | Freq. (BC)  cases/contr. | P  (BC) | Freq. (OC)  cases/contr. | P  (OC) | Freq.  (HMP) |
| --- | --- | --- | --- | --- | --- | --- | --- | --- | --- | --- | --- |
| **1** | g | a | - | - | a | c | 0.32/0.31 | 0.62 | 0.32/0.31 | 0.61 | 0.33 |
| **2** | t | g | - | - | a | t | 0.30/0.30 | 0.81 | 0.30/0.31 | 0.83 | 0.32 |
| **3** | t | g | - | - | a | c | 0.16/0.16 | 0.65 | 0.17/0.16 | 0.71 | 0.13 |
| **4** | t | g | a | - | g | t | 0.13/0.13 | 0.56 | 0.13/0.13 | 0.34 | 0.07 |
|  |  |  | g |  |  |  |  |  |  |  | 0.07 |
| **5** | t | a | - | g | a | c | 0.08/0.09 | 0.28 | 0.08/0.08 | 0.80 | 0.03 |
|  |  |  |  | t |  |  |  |  |  |  | 0.02 |
